# Intramedullary Nail vs. Plate Fixation for Pathological Humeral Shaft Fracture: An Updated Narrative Review and Meta-Analysis of Surgery-Related Factors

**DOI:** 10.3390/jcm13030755

**Published:** 2024-01-28

**Authors:** Bing-Kuan Chen, Ting-Han Tai, Shu-Hsuan Lin, Kuan-Hao Chen, Yu-Min Huang, Chih-Yu Chen

**Affiliations:** 1 School of Medicine, College of Medicine, Taipei Medical University, Taipei 11031, Taiwan; 22271@s.tmu.edu.tw (B.-K.C.); 19167@s.tmu.edu.tw (T.-H.T.); 19267@s.tmu.edu.tw (S.-H.L.); 2 Division of General Medicine, Department of Medical Education, Shuang Ho Hospital, Taipei Medical University, New Taipei City 23561, Taiwan; 3 Department of Orthopedics, Shuang Ho Hospital, Taipei Medical University, New Taipei City 23561, Taiwan; 14276@s.tmu.edu.tw (K.-H.C.); 08591@s.tmu.edu.tw (Y.-M.H.); 4 Graduate Institute of Biomedical Materials and Tissue Engineering, College of Biomedical Engineering, Taipei Medical University, Taipei 11031, Taiwan; 5 International Ph.D. Program in Biomedical Engineering, College of Biomedical Engineering, Taipei Medical University, Taipei 11031, Taiwan

**Keywords:** plate, intramedullary nail, internal fixation, pathological humeral shaft fractures, bone metastasis, metastatic lesion, meta-analysis

## Abstract

(1) **Background:** Pathological humeral shaft fracture (PHSF) is a frequently observed clinical manifestation in the later stages of tumor metastasis. Surgical interventions are typically recommended to alleviate pain and restore functionality. Intramedullary nail fixation (INF) or plate fixation (PF) is currently recommended for the treatment of PHSF. However, there is still no standard for optimal surgical treatment. Thus, we conducted a meta-analysis comparing the clinical outcomes of INF with PF for PHSF treatment. (2) **Methods:** We conducted searches in databases, such as Scopus, EMBASE, and PubMed, for studies published prior to May 2023. In total, nine studies with 485 patients were reviewed. (3) **Results:** There were no significant differences noted in the incidence of fixation failure, local recurrence, wound complication or overall complication. However, the INF group demonstrated a significantly lower incidence of postoperative radial nerve palsy than the PF group (OR, 5.246; 95% CI, 1.548–17.774; *p* = 0.008). A subgroup analysis indicated that there were no statistically significant differences in fixation failure or local recurrence among subgroups categorized by the design of intramedullary nail. (4) **Conclusions:** Considering the short life expectancy of end-stage patients, the choice of surgical method depends on the patient’s individual condition, fracture and lesion patterns, the surgeon’s experience, and comprehensive discussion between the surgeon and patient.

## 1. Introduction

Pathological fracture is a frequently encountered clinical presentation in patients with later stages of tumor metastasis. Once solid tumor metastasis to the skeleton, bone remodeling sequences are disrupted, leading to the presentation of discrete osteolysis, diffuse osteopenia, osteoblastic lesions or a combination of these. Pathological humeral shaft fracture (PHSF), the second most common manifestation of bone metastasis preceded only by femur, contributes to a substantial portion of cases involving either current pathological fractures or those that are impending, with reported incidences ranging from 16% to 39% [1,2,3]. Although the humerus is not a weight-bearing bone, the development of a pathological fracture in this region can still result in significant, unrelenting arm or shoulder pain during periods of rest, especially at night, which often does not respond to analgesic interventions. Consequently, this may lead to restricted hand functionality and a consequential reduction in the patient’s overall quality of life [4]. In order to stabilize the fracture and return mobility with full weightbearing as soon as possible, surgical intervention becomes a necessity [5,6]. However, the most effective surgical technique for PHSF remains controversial.

There are several surgical techniques available for PHSF stabilization including intramedullary nail, plate, and segmental prosthesis [1,7]. Traditionally, segmental prosthesis was limited to patients with solitary lesions and with bony destruction involving adjacent joints. Segmental prosthesis has several advantages including a shorter required hospital stay, early mobilization and the ability to tolerate chemotherapy and radiotherapy [8]. However, the usage of segmental prosthesis is declining due to high complication rates involving mechanical failure and aseptic loosening [5,9,10,11]. Therefore, we excluded studies concerning segmental prosthetic replacement in this study, and focused on intramedullary nail fixation (INF) and plate fixation (PF). INF and PF are commonly applied strategies in the treatment for PHSF nowadays. Both methods, however, have both advantages and drawbacks.

**Figure 1:**
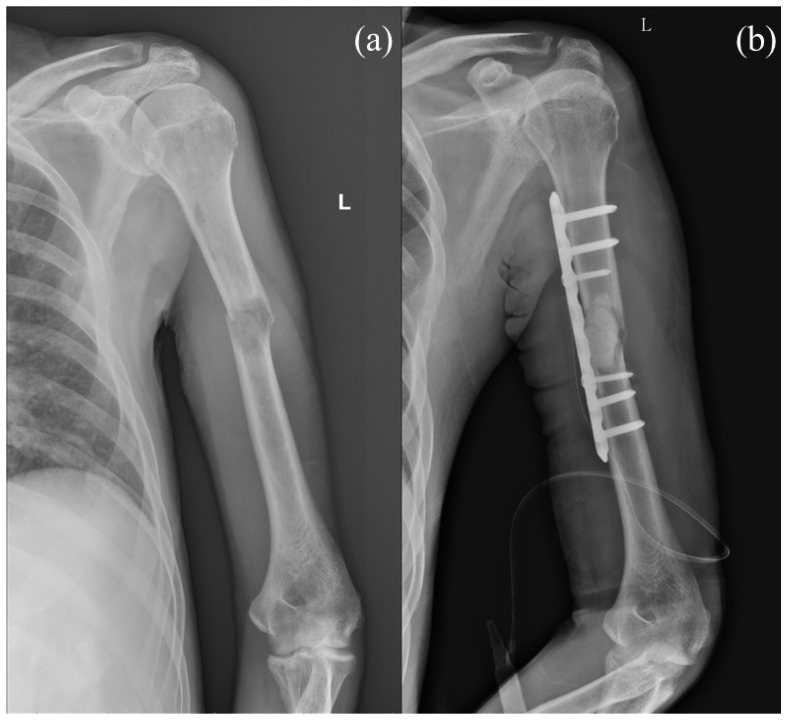
(**a**) Preoperative image of a case with diffuse liver tumor and left pathological humeral shaft fracture. (**b**) Plate fixation with cement augmentation was performed for the pathological humeral shaft fracture. Letter “L” in the figure are referred to as left.

PF is commonly used in patients with solitary lesions because it provides a direct approach to the lesion, enabling the surgeon to explore the border of the tumor while preserving shoulder function with a better operating view, maintaining the integrity of the rotator cuff and lessening tumor burden (Figure 1) [5]. However, concerns regarding PF include potential postoperative radial nerve palsy, a larger incision leading to increased wound complications, and the relatively limited fixation length [5,6].

**Figure 2:**
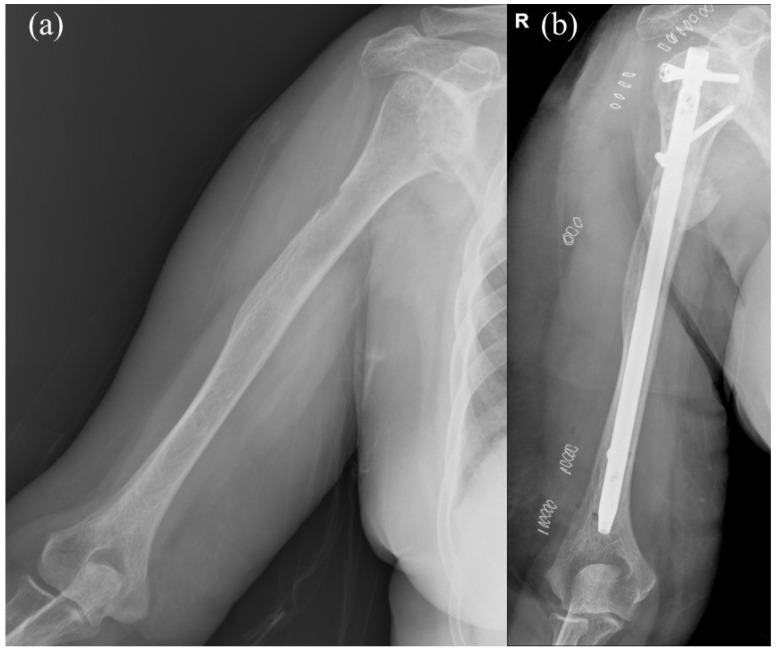
(**a**) Preoperative image of a case with impending pathological fracture of right humeral shaft. (**b**) Intramedullary nail fixation with cement augmentation and bone curettage were performed for the impending pathological humeral shaft fracture. Letter “R” in the figure are referred to as right.

On the other hand, INF is a minimally invasive surgery with small incision wounds and is the favored surgical techniques for PHSF due to lower blood loss, decreased surgical time and better biomechanical stability (Figure 2). However, shoulder complications have been reported with INF, including rotator cuff damage, nail protrusion and shoulder impingement, leading to deteriorating quality of life for late-stage cancer patients.

There are substantial disparities in patient lifespan, origins of metastatic tumors, underlying diseases, and treatment modalities among PHSF patients, and there is a scarcity of individualized literature dedicated to pathologic humeral shaft fractures. As a result, there is no clear evidence, systematic review or meta-analysis comparing the clinical outcomes regarding fixation failure, local recurrence, wound complications and postoperative radial nerve palsy between INF and PF for patients with PHSF. Accordingly, we conducted a meta-analysis to compare the outcomes of the INF and PF methods in the treatment of PHSF and performed a literature review regarding the economic cost and appropriate surgical candidates for each treatment.

## 2. Materials and Methods

This study adhered to the guidelines outlined in the Preferred Reporting Items for Systematic Reviews and Meta-analyses statement (PRISMA). This meta-analysis was registered in the International Prospective Register of Systematic Reviews, curated by the National Institute for Health Research (registration no. CRD42023411131).

### 2.1. Study Design and Identification of Eligible Studies

Two main investigators (T.-H.T. and B.-K.C.) conducted a thorough search for pertinent studies published prior to May 2023 across the PubMed, Embase, and Scopus databases independently. The following keywords were searched during the process: ((“Humerus” OR “Humeral Diaphysis” OR “Humeral Shaft”) AND (“metastasis” OR “pathologic” OR “metastatic”)) AND (“surgical” OR (“intramedullary nailing” AND “plate”)). In order to perform a more comprehensive and accurate analysis, we screened the reference lists of pertinent studies and utilized the “related articles” feature in PubMed to further explore relevant research.

### 2.2. Inclusion and Exclusion Criteria

Randomized control trials (RCTs), prospective cohort studies, and retrospective cohort studies that evaluated the outcomes of INF and PF in treatment for PHSF were eligible for this meta-analysis. Studies were required to clearly report the inclusion and exclusion criteria of enrolled patients. Furthermore, details of the surgical procedures, definitions and evaluations of outcome parameters, and study endpoint should be elaborated in the study. To focus the topic, we excluded studies that included (1) patients without pathological-related humeral fracture, (2) patients who had previously undergone segmental prosthesis fixation, (3) duplicate patient cohorts, (4) paediatric cohorts, and (5) insufficient data regarding outcomes of the diaphysis lesion.

### 2.3. Data Extraction

Two main investigators (T.-H.T. and B.-K.C.) independently identified and reviewed the relevant studies, and extracted the baseline and follow-up data from either the text, datasets, figures or tables. The extracted data included the name of the first author, year of publication, country, sample size, participants’ ages, gender, pathological fracture pattern, surgical intervention, and peri-operative outcomes of both patients with PHSF receiving INF or PF. Decisions recorded individually by the investigators were compared, and disagreements were resolved by the third reviewer (C.-Y.C.).

### 2.4. Methodological Quality Appraisal

Two main investigators (T.-H.T. and B.-K.C.) independently assessed the methodological quality of the included observational studies using the Newcastle–Ottawa Scale (NOS).

The NOS consists of three domains: selection (four items), comparability (one item), and outcome (three items). The maximum rating on the NOS is nine stars, consisting of the comparability item, which can be assigned up to two stars, while the remaining items can be assigned one star at most.

A study achieving a score of 8 or 9 stars was categorized as high quality, while a score of 5 to 7 stars indicated moderate or low quality. Studies with fewer than 5 stars were considered poor quality. In cases of discrepancies during the assessment and rating of studies, any disagreements were resolved through discussion with the third reviewer (C.-Y.C.).

### 2.5. Outcomes

The primary outcomes of interest were fixation failure rate and local recurrence rate in patients with PHSF undergoing INF or PF. Furthermore, postoperative radial nerve palsy rate, wound complication rate, and overall complication rate were also examined.

### 2.6. Statistical Analyses

The extracted data were meta-analyzed in May 2023 using Comprehensive Meta-Analysis Software (Version 3.3.070, Biostat, Englewood, NJ, USA). Continuous variables were presented as means with standard deviations (SDs), and the differences in continuous variables were measured using standardized mean difference and 95% confidence intervals (CIs). Dichotomous variables were extracted and transformed into percentages due to the lack of absolute numbers in some included studies. Difference in dichotomous variables were measured using odds ratio (OR) and 95% CIs. Statistical significance was determined at a *p*-value of <0.05. We performed subgroup analysis based on implant type (interlocking intramedullary nail and non-interlocking intramedullary nail). A *p*-value less than 0.10 indicated a significant difference between subgroups. Heterogeneity among the studies included in the analysis was assessed using the chi-squared test, Cochran’s Q test, and I^2^ test, with significance established at *p* < 0.1 for the Cochran’s Q test and I^2^ values exceeding 50% [11]. In the *z*-test for equivalence, significance was indicated by *p* < 0.05. Evaluation of publication bias was conducted using Egger’s statistical test, and the analysis was performed with the Comprehensive Meta-Analysis Software (Version 3.3.070, Biostat, Englewood, NJ, USA).

## 3. Results

**Figure 3:**
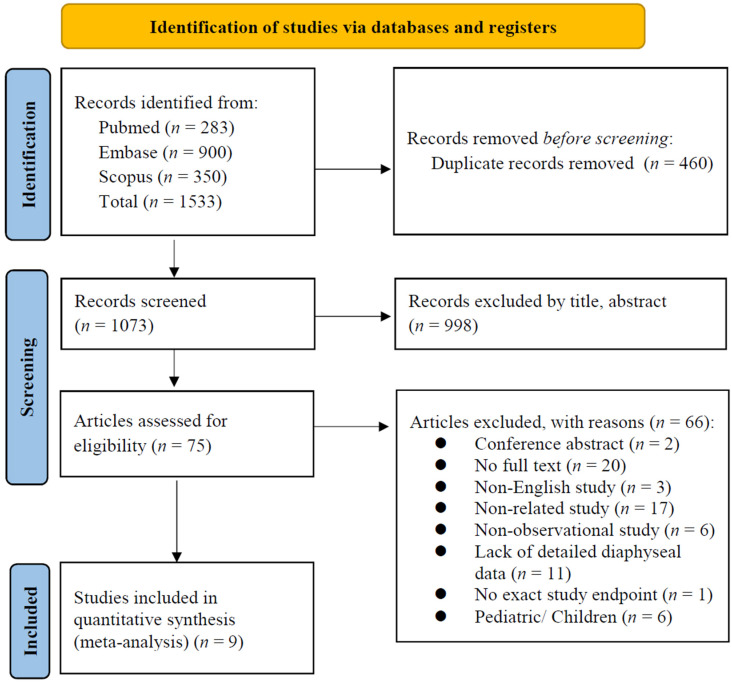
PRISMA flowchart of the selection of included studies.

Figure 3 presents the detailed steps of the article selection process. A total of 1533 studies were identified through the initial searches of the databases (PubMed, Embase and Scopus). After removing 460 duplicated studies, 998 studies were also excluded after examining the titles and abstracts. The remaining 75 studies underwent full-text review. Of these studies, 66 were excluded for the following reasons: 2 studies were conference abstracts, 20 studies had no available full text, 3 were non-English studies, 17 were non-related studies, 6 were not observational studies, 11 lacked detailed data regarding diaphysis, 1 did not report an exact study endpoint, and 6 were paediatric only studies. The remaining nine studies were included in the meta-analysis [1,3,9,12,13,14,15,16,17].

The characteristics of the included studies are reported in Table 1 and the available treatment details are listed in Table 2. All the nine included studies were observational comparative studies [1,3,9,12,13,14,15,16,17]. Five of the studies reported exact follow-up duration, but only two studies reported exact mean follow-up duration for patients with bone metastasis of humerus diaphysis [9,15]. Six studies were European, two were North American, and one was Asian. In total, our study comprised a population of 485 participants, and the mean age was 64.9 years, with 54.7% being female in studies providing diaphysis-limited data.

The methodological quality of the included studies was assessed using the NOS, and the results are presented in Supplementary Table S1. All the studies were assessed as moderate or low quality, mainly due to a lack of comparability between cohorts and insufficient follow-up time.

### 3.1. Quantitative Data Synthesis (Meta-Analysis)

#### 3.1.1. Fixation Failure Rate

**Figure 4:**
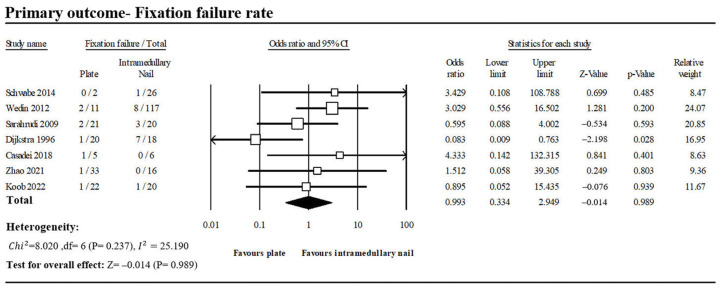
Forest plot of seven studies comparing fixation failure between PF and INF groups [1,3,9,13,14,15,17].

Fixation failure was defined as a refracture of the humerus, loosening of the screws, instability, or any reoperation after the primary surgical procedure. Seven of the included studies reported cases of fixation failure following PF and INF treatment for PHSF during follow-up (Figure 4) [1,3,9,13,14,15,17]. In total, 8 of 114 patients in the PF group and 20 of 223 patients in the INF group were noted with fixation failures. Pooled analysis revealed no significant difference between the PF group and the INF group (OR, 0.993; 95% CI, 0.334–2.949; *p* = 0.989). Low heterogeneity was observed across the analyzed studies (*I*^2^: = 25.19%, *p* = 0.237).

#### 3.1.2. Local Recurrence Rate

**Figure 5:**
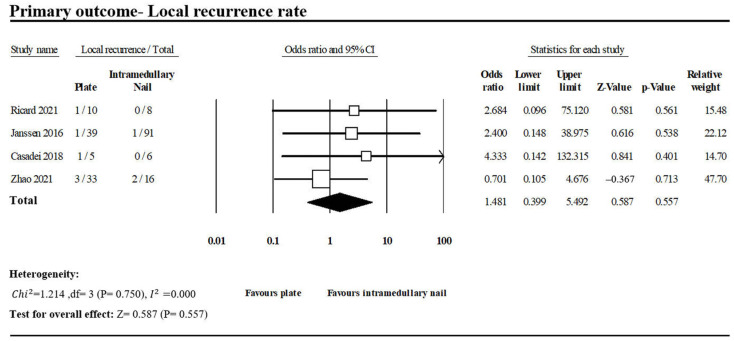
Forest plot of four studies comparing local recurrence between PF and INF groups [9,12,14,16].

Four of the included studies reported cases of local recurrence following PF and INF treatment for PHSF during follow-up (Figure 5) [9,12,14,16]. After pooling the extracted data, 6 of 87 patients in the PF group and 3 of 121 patients in the INF group were found developing local recurrence. No significant difference in local recurrence rate between the PF group and the INF group (OR, 1.481; 95% CI, 0.399–5.492; *p* = 0.557). No heterogeneity was found (*I*^2^: 0%, *p* = 0.750).

#### 3.1.3. Postoperative Radial Nerve Palsy

**Figure 6:**
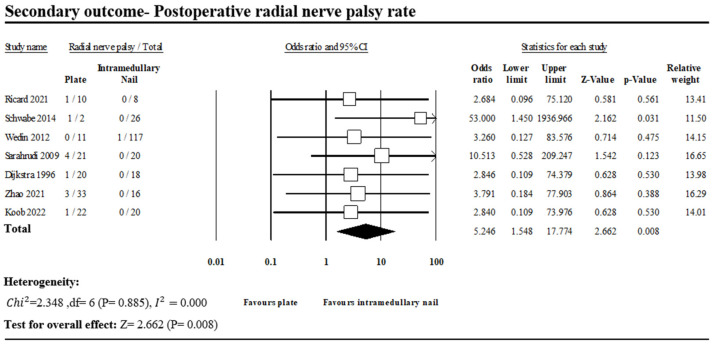
Forest plot of seven studies comparing postoperative radial nerve palsy between PF and INF groups [1,3,9,13,15,16,17].

Cases of postoperative radial nerve palsy were reported in seven of the included studies (Figure 6) [1,3,9,13,15,16,17]. In total, 11 of 119 patients in the PF group had postoperative radial nerve palsy against 1 of 225 patients in the INF group, with a statistically significant difference (OR, 5.246; 95% CI, 1.548–17.774; *p* = 0.008). No heterogeneity was observed across the analyzed studies (*I*^2^: 0%, *p* = 0.885).

#### 3.1.4. Wound Complication Rate

**Figure 7:**
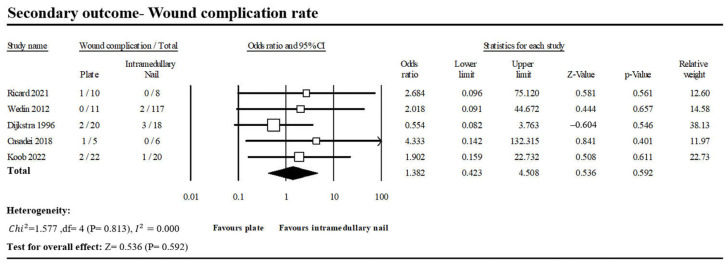
Forest plot of five studies comparing wound complication between PF and INF groups [1,13,14,15,16].

Wound complication was defined as postoperative wound infection, wound hematoma or wound dehiscence after the operation. Five of the included studies reported 6 of 68 patients in the PF group and 6 of 169 patients in the INF group with wound complications (Figure 7) [1,13,14,15,16]. There was no significant difference between the two groups (OR, 1.382; 95% CI, 0.423–4.508; *p* = 0.592). No heterogeneity was observed across the analyzed studies (*I*^2^: 0%, *p* = 0.813).

#### 3.1.5. Overall Complication Rate

**Figure 8:**
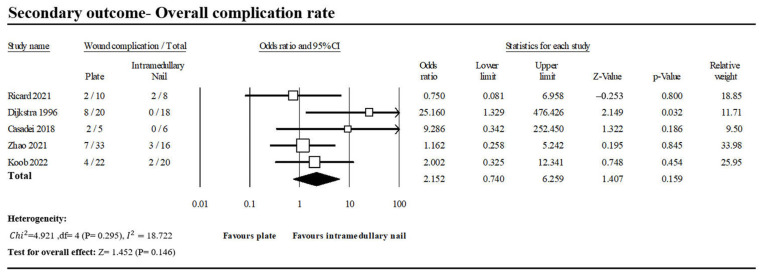
Forest plot of five studies comparing overall complication between PF and INF groups [1,9,14,15,16].

Five of the included studies reported 23 of 90 patients in the PF group and 7 of 68 patients in the INF group with complications (Figure 8) [1,9,14,15,16]. There was no significant difference between the two groups (OR, 2.152; 95% CI, 0.740–6.259; *p* = 0.295). Low heterogeneity was observed across the analyzed studies (*I*^2^: 18.72%, *p* = 0.146).

### 3.2. Interlocking Intramedullary Nail Subgroup Analysis

**Figure 9:**
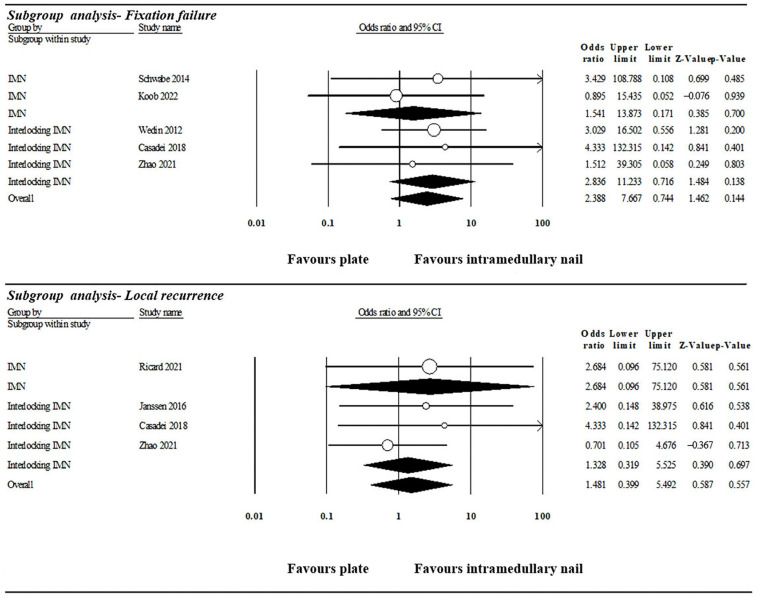
Subgroup analysis of fixation failure based on the design of intramedullary nail. IMN: intramedullary nail [9,12,13,14,15,16,17].

Four studies presented data which were used in the subgroup analysis of pathological humerus diaphysis fracture treated by ORIF with interlocking intramedullary nail (Figure 9) [9,12,13,14]. Subgroup analyses according to the type of intramedullary nail showed no subgroup being significantly better compared with plate fixation in the incidence of fixation failure and local recurrence.

### 3.3. Publication Bias

**Figure 10:**
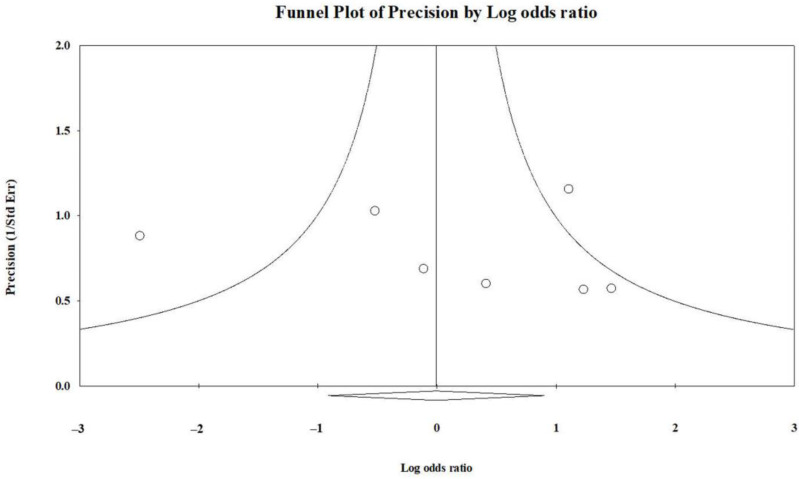
Funnel plot depicting the comparison of fixation failure incidence between the INF and PF groups.

The funnel plot of the studies comparing the incidence of fixation failure between the INF and PF groups for PHSF is presented in Figure 10. The plot reveals a symmetric distribution. No significant publication bias was detected through Egger’s statistical test (*t* value = 0.331, 2-tailed *p* value = 0.754).

## 4. Discussion

Various surgical options, including INF, PF, and prosthesis replacement, are applied for PHSF stabilization according to the location of the lesions, amount of bony involvement and disease response to systemic treatment [2,18,19]. Although prosthetic arthroplasty is reported with rigid fixation for patients with solitary lesions and large cortical destruction [9], concerns regarding aseptic loosening, slow functional recovery, subsequent rehabilitation period and economic burden have resulted in declined usage for PHSF treatment [5,10,11]. Hence, we excluded studies with intercalary prosthetic replacement and focused on the comparisons of INF and PF. The findings provide valuable insights into the nuanced outcomes associated with each technique.

### 4.1. Surgical and Clinical Features of Plate Fixation

PF provides better access to the lesion site than INF through the creation of larger incisions [9,15,16], which also lead to significantly increased blood loss and prolonged operation time [3,9,15]. According to the literature, patients with pathological humeral shaft fractures require an average of 153–174 min for PF surgery, a notably longer duration than the average of 48–136 min for the INF group [3,9,15,20].

In addition, the PF group revealed non-significantly superior performance in fixation failure (PF vs. INF: 7% vs. 9%; OR, 0.993; *p* = 0.989) than the INF group. However, only one of the included studies revealed significant clinical advantages for PF [1], while the remaining studies did not comprehensively support PF or failed to show significant differences. 

Furthermore, other studies have demonstrated a significantly higher incidence in postoperative radial nerve damage in the PF group, influencing quality of life in terminal-stage patients [21,22]. These findings were also consistent with our study. In the current study, the PF group contributed to a significantly higher incidence of postoperative radial nerve palsy (PF vs. INF: 9.2% vs. 0.4%; OR, 5.246; *p* = 0.008).

### 4.2. Surgical and Clinical Features of Intramedullary Nail Fixation

INF was reported to have better load-sharing ability, less blood loss during operation and a lower incidence of postoperative radial nerve damage. Moreover, intramedullary nail was proved to provide sufficient stability in impending fractures and pathological fractures which occur in the area located 2–3 cm distal to the greater tuberosity and 5 cm proximal to the olecranon fossa [6]. Furthermore, INF has benefits in patients with metastatic lesions with bleeding tendency, such as those originating from the liver, kidney, or thyroid, due to the smaller incision required [22,23,24]. As a consequence of these advantages, INF is considered a more favorable surgical methods than PF for PHSF treatment [5,13,25]. 

In the current study, the INF group demonstrated non-significantly superior performance over the PF group in local recurrence (PF vs. INF: 6.9% vs. 2.5%; OR, 1.481; *p* = 0.557). 

We have two interpretations for this trend. First, local recurrence is more associated with the effectiveness of systemic treatment, rather than surgical management, which primarily serves as symptom control. Secondly, making an incision around the tumor site poses a greater risk of tumor seeding and contamination compared with using an intramedullary nail inserted at a distance from the tumor.

Due to the smaller incisions, the INF group also demonstrated marginally better outcomes in terms of wound complications (PF vs. INF: 8.8% vs. 3.6%; OR, 1.382; *p* = 0.592), overall complications (PF vs. INF: 25.6% vs. 10.3%, OR, 2.152; *p* = 0.295), reduced operation duration (Standardized mean difference (SMD) 1.436, 95% CI 0.190 to 2.683; I^2^ = 89.6%), and diminished blood loss (Standardized mean difference (SMD) 3.841, 95% CI −1.207 to 8.990; I^2^ = 97.4%) compared with the PF group in this study. However, significant heterogeneity was observed in the pooled analysis, warranting careful consideration and interpretation of these findings.

### 4.3. Applications of Postoperative Radiotherapy

Insufficient clinical evidence raises debates about the application of radiotherapy in the postoperative management of long bone metastases [26]. Previous studies have highlighted certain risk factors for fixation failure, such as extremity bone metastasis as opposed to spine metastasis, limited coverage of surgical hardware by radiotherapy, and extended intervals between surgery and radiotherapy delivery, indicating a crucial role of radiotherapy [27]. 

For pathological humeral shaft fracture, Dijkstra et al. suggested avoiding routine postoperative radiotherapy due to transient local osteoporosis effects in patients treated with plates and bone cement. Instead, they recommended routine adjuvant radiotherapy for comprehensive local tumor control in patients treated with intramedullary nails [1,28]. In the present study, there was no detailed information available regarding the treatment modality for postoperative radiotherapy for either the INF or PF group. Only two of the studies included mentioned using routine postoperative therapy, yet no significant differences in fixation failure or local recurrence were noted across these studies [9,13]. Ofluoglu et al. utilized closed intramedullary locking nailing followed by early postoperative radiation in 24 cases of pathological humeral shaft fractures. Their findings revealed that 90% of the patients experienced pain resolution one month after the surgery, and they achieved 64% of normal upper extremity function, as evaluated by the Musculoskeletal Tumor Society upper extremity scoring system, with a mean follow-up duration of 17 months. The above findings emphasize the potential for symptom relief via smaller incisions with intramedullary nails and subsequent radiotherapy [29].

### 4.4. Shoulder Complications for INF

INF is prone to cause shoulder complications including rotator cuff damage, nail protrusion and shoulder impingement [4,23,30]. Various factors were considered as the contributing factors for shoulder complications, including the telescopic effect, direct damage caused by the entry point, incomplete embedding of the nail end at surgery and impingement of the head of proximal interlocking screw [31]. To avoid rotator cuff damage and shoulder impingement, retrograde insertion of an intramedullary nail is considered a better method than antegrade insertion because it avoids insertion via the footprint of the supraspinatus and humeral head [31]. However, Heinsen et al. asserts that the antegrade insertion of an intramedullary nail does not correlate with an elevated risk of shoulder complications, provided that the repair of the supraspinatus tendon and the appropriate embedding of the nail end in the humeral head are executed accurately [32]. Furthermore, a recent retrospective study has demonstrated favorable functional and radiographic outcomes without shoulder complications when employing percutaneous antegrade intramedullary nailing with a long straight dynamic locking nail that penetrates the supraspinatus muscle zone but avoids the footprint [33]. 

### 4.5. Surgical Costs for Both Methods

Surgical costs involve direct and indirect costs, and should also be taken into consideration due to patients’ short life expectancy and the potential costs of adjuvant radiotherapy. Direct fixed costs are essential non-variable expenses inherent in the operation of a hospital, whereas direct variable costs include expenditures like medications, medical tests, or surgical equipment. Indirect costs, which are not directly linked to patient care, pertain to non-revenue-producing areas of the hospital, such as the financial services department and information technology [34].

A previous analysis conducted in America compared the total direct costs of the surgical encounter for INF and PF groups with adjustment for inflation, and demonstrated that the INF group incurred approximately 20% higher costs than the PF group, with statistical significance [35]. Regarding the indirect costs, there were no significant differences in operation room utilization costs or post-anesthesia care unit utilization costs between the two groups. However, the cost of implants, surgical expenses, hospitalization fees, and the various healthcare insurance reimbursement systems are related to each country’s circumstances. Additionally, the costs associated with operating room usage, anesthesia time, surgical time, and other factors are also influenced by the equipment of different hospitals and the experience of surgeons. Therefore, thorough investigations of cost based on the specific conditions in each country are warranted.

### 4.6. Similar Studies

A recent retrospective study comparing clinical outcomes between cemented plate fixation and INF for proximal humerus pathological fracture revealed that INF group demonstrated a significantly greater reduction in VAS score (median (IQR): 7.0 (6.0–8.0) vs. 6.0 (5.0–7.0), *p*  =  0.010) than cemented plate group at one month after the operation. Furthermore, an equivalent performance in Musculoskeletal Tumor Society rating scale and Karnofsky performance status scale three months after the operation were also noted, indicating that excellent treatment efficacy in pain relief and functional status could be achieved with the intramedullary nail implant [36]. With continuous innovation in the design of nail implants, their limitations in providing rigid fixation and shoulder complications have been gradually addressed. However, based on the numerous advantages mentioned above, the utilization of intramedullary nailing for the treatment of traumatic, osteoporotic, pathological humeral shaft fractures is on the rise [36].

### 4.7. Recommended Surgical Approach Based on Different Conditions

Given the limited life expectancy and complex conditions associated with pathological fractures, we aim to utilize this meta-analysis to identify the treatment modality that can provide the maximum benefit to end-of-life patients in the short term. Several indications have been proposed according to the features of the lesions [4,9]. Furthermore, Lin et al. categorized the recommendations of surgical methods according to features of the lesions and the pursuit of long-term or short-term care (Table 3) [37].

However, in cases in which the tumor is extensive, infiltrating bone and affecting soft tissues, tumor excision coupled with plate fixation would be more appropriate. Additionally, if skip lesions prevent the plate from offering complete stability, the application of a nail would still be the preferable option.

In summary, the optimal operative treatment strategies for PHSF depends on the patient’s individual condition and the surgeon’s experience, as well as the anatomical location, pathological features of the lesions and availability of resources.

## 5. Limitations

There are several potential limitations in our studies. First, no randomized controlled trial but only two prospective cohort studies and seven retrospective studies were included in current study, leading to potential selection bias and the inability to determine causality. In addition, only five of the included studies focused on diaphyseal lesions, resulting in poor control of confounding factors after pooling of the extracted data. Furthermore, the sample sizes of the included studies were relatively small. In addition, the length of follow-up duration in each article was not fixed or available because of the high mortality of metastatic disease and the limited information regarding diaphyseal lesions, which made it hard to examine the long-term outcomes of the implants and interpret the results accurately. Finally, we were almost not able to conduct the subgroup analysis due to the limited literature providing a comprehensive description of diaphysis lesions, along with the complex and diverse nature of the condition. Fortunately, the heterogeneity of the primary outcomes in this study are not high, which enhances the credibility of the results. Further randomized controlled studies with larger sample sizes are warranted to comprehensively compare the treatment efficacy between INF and PF for patients with PHSF.

## 6. Conclusions

Both intramedullary nailing and plating are safe and effective surgical methods for treating metastatic lesions in humeral shaft fractures. In this study, there were no significant differences noted in the incidence of fixation failure, local recurrence, wound complication or overall complication. However, the INF group demonstrated a significantly lower incidence of postoperative radial nerve palsy than the PF group. Considering the short life expectancy and complexity of end-stage patients, the choice of surgical method depends on the patient’s individual condition, the fracture and lesion patterns and the surgeon’s experience. Therefore, comprehensive discussion between surgeons and patients and shared decision-making are essential. 

## Figures and Tables

**Table 1:** Baseline characteristics of the included studies.

Author (Year)	Country	Design	Sample Size (n)	Mean Age (Years)	Female (n (%))	Endpoint	Follow-Up Duration (Months) (Mean ± SD) (Median, Range)	Outcome
Dijkstra et al. (1996) [1]	Netherlands	Retrospective cohort study	PF: 20 INF: 18	PF: 63 INF: 68	26 (70%)	At least six months/Death	4.8 (mean)	(1) (3) (4) (5)
Sarahrudi et al. (2009) [3]	Austria	Retrospective cohort study	PF: 21 INF: 20	PF: 70.2 INF: 66.3	26 (63.4%)	Implant failure/Death	Not-mentioned	(1) (3)
Wedin et al. (2012) [13]	Sweden, Denmark, Norway	Prospective cohort study	PF: 11 INF: 117	No diaphysis-limited data	No diaphysis-limited data	Implant failure/Death	8, 0–97 (No diaphysis-limited data)	(1) (3) (4)
Schwabe et al. (2014) [17]	Germany	Retrospective cohort study	PF: 2 INF: 26	No diaphysis-limited data	No diaphysis-limited data	Implant failure/Death	Not-mentioned	(1) (3)
Janssen et al. (2016) [12]	America	Retrospective cohort study	PF: 39 INF: 91	No diaphysis-limited data	No diaphysis-limited data	Implant failure/Death	4, 0–120 (Not diaphysis-limited data)	(2)
Casadei et al. (2018) [14]	Italy	Retrospective cohort study	PF: 5 INF: 6	Total: 66.6	3 (25%)	At least 2 months from surgery	22 (mean) (Not diaphysis-limited data)	(1) (2) (4) (5)
Ricard et al. (2021) [16]	Canada	Prospective cohort study	PF: 10 INF: 8	PF: 61.2 INF: 70.25	8 (44.4%)	At least 52 weeks of follow-up or/Death	Not-mentioned	(2) (3) (4) (5)
Zhao et al. (2021) [9]	China	Retrospective cohort study	PF: 33 INF: 16	PF: 62.1 INF: 61.9	30 (48%)	Implant failure/Death	20.83 ± 18.4	(1) (2) (3) (5)
Koob et al. (2022) [15]	Germany	Retrospective cohort study	PF: 22 INF: 20	Total: 64.2	16 (38.09)	Implant failure/Death	8.5 ± 15.4	(1) (3) (4) (5)

PF: Plate fixation group; INF: Intramedullary nail fixation group; (1) Fixation failure rate; (2) Local recurrence rate; (3) Postoperative radial nerve palsy rate; (4) Wound complication rate; (5) Overall complication rate.

**Table 2:** Treatment details of the included studies.

Author (Year)	Implant Details	Preoperative Radiotherapy	Postoperative Radiotherapy	From Diagnosis of Primary Tumor to Humeral Metastasis (Months) (Mean ± SD) (Median/Mean, Range)	From Diagnosis of Primary Tumor to Surgery (Months) (Mean ± SD) (Median, Range)
Dijkstra et al. (1996) [1]	PF: Plate with bone cement INF: Antegrade nail without bipolar static locking for 2 cases; Interlocking nail with antegrade procedure for 9 cases, Retrograde operation in 7 cases	33% of the patient cohort (17 Gy)	25% of the PF group, and all the INF groups	Not-mentioned	Not-mentioned
Sarahrudi et al. (2009) [3]	PF: Dynamic compression plate in 18 patients, locking compression plate in 2 patients and Y-plate in 1 patient. All augmented with cement INF: Unreamed humeral nail in 15, a Seidel nail in 3, and an AR-Nail in 1 patient.	Not-mentioned	Not-mentioned	Not-mentioned	Not-mentioned
Wedin et al. (2012) [13]	PF: Plate without bone cement INF: Interlocked intramedullary nail	No diaphysis-limited data	Routinely	10, 0–288 (median, range) (Not diaphysis-limited)	23, 0–289 (median, range) (Not diaphysis-limited)
Schwabe et al. (2014) [17]	PF: Locking-compression plate with bone cement INF: Intramedullary nail	No diaphysis-limited data	No diaphysis-limited data	14.5, 0–173 (mean, range)	21.4, 0–173 (median, range)
Janssen et al. (2016) [12]	PF: Plate-screw fixation with bone cement in 19 cases; Plate-screw fixation without bone cement in 20 cases INF: Interlocked intramedullary nail	No diaphysis-limited data	Not-mentioned	Not-mentioned	Not-mentioned
Casadei et al. (2018) [14]	PF: Plate with bone cement INF: Interlocked intramedullary nail	Not-mentioned	33% of the patient cohort	Not-mentioned	Not-mentioned
Ricard et al. (2021) [16]	PF: Plate with bone cement in 9 cases; plate without bone cement in 1 case INF: Intramedullary nail with cement in 2 cases, without cement in 6 cases	16.7% of the patient cohort received	5.6% of the patient cohort received	Not-mentioned	Not-mentioned
Zhao et al. (2021) [9]	PF: Plate with bone cement INF: Interlocked intramedullary nail	31.7% of the patient cohort received	4 weeks postoperatively	13.5 ± 25.6	Not-mentioned
Koob et al. (2022) [15]	PF: Plate with bone cement INF: Intramedullary nail with bone cement	Not-mentioned	Not-mentioned	Not-mentioned	Not-mentioned

PF: Plate fixation group; INF: Intramedullary nail fixation group.

**Table 3:** Surgery recommendations according to characteristics of the disease.

Features of PHSF	Short-Term Care	Long-Term Care
Large solitary lesion	PF	PF
Impending fracture or multiple lesions	INF	INF

Abbreviations: PHSF, pathological humeral shaft fracture; PF, plate fixation; INF, intramedullary nailing fixation.

## Data Availability

Data are available on request.

## Supplementary Materials

The following supporting information can be downloaded at: https://www.mdpi.com/article/10.3390/jcm13030755/s1. Table S1: The Newcastle–Ottawa Scale (NOS) of the included studies.
