# Supplementary material for: Intramedullary Nail vs. Plate Fixation for Pathological Humeral Shaft Fracture: An Updated Narrative Review and Meta-Analysis of Surgery-Related Factors
Source: J Clin Med. 2024 Jan 28;13(3):755. doi: 10.3390/jcm13030755 (PMC10856436; doi:10.3390/jcm13030755)
Supplement: Supplementary file 1 [file jcm-13-00755-s001.zip › jcm-2767221-supplementary.pdf]

Table S1: The Newcastle-Ottawa Scale (NOS) of the included studies										
Study	Selection				Comparability		Outcome			Total
	Representative of the exposed cohort	Selection of external control	Ascertain-ment of exposure	Outcome of interest not present at the start of the study	Comparability of cohorts		Assessment of outcomes	Sufficient follow-up time	Adequacy of follow-up	
					Main factor	Additional factor				
Dijkstra et al. [1996] [1]	+	+	+	+	-	-	+	+	+	7
Sarahrudi et al. [2009] [3]	+	+	+	-	-	-	+	-	+	5
Wedin et al. [2012] [13]	+	+	+	+	-	-	+	-	+	6
Schwabe et al. [2014] [17]	+	+	+	+	-	-	+	-	+	6
Janssen et al. [2016] [12]	+	+	+	+	-	-	+	+	+	7

Casadei et al. [2018] [14]	+	+	+	+	-	-	+	+	+	7
Zhao et al. [2021] [9]	+	+	+	+	-	-	+	+	+	7
Ricard et al. [2021] [16]	+	+	+	+	-	-	+	+	+	7
Koob et al. [2022] [15]	+	+	+	+	-	-	+	+	-	6
